# Application of carbon nanoparticles combined with refined extracapsular anatomy in endoscopic thyroidectomy

**DOI:** 10.3389/fendo.2023.1131947

**Published:** 2023-06-02

**Authors:** Zheng Wang, Hongguang Bo, Yufa Xu, Zilin Bi, Zhaocai Yin, Changsheng Yu, Enxi Luo, Xiaomeng Shi, Bin Chen, Yabing Wang, Rui Sha

**Affiliations:** Department of Thyroid and Breast Surgery, Yijishan Hospital, First Affiliated Hospital of Wannan Medical College, Wuhu, Anhui, China

**Keywords:** extracapsular anatomy, carbon nanoparticles, endoscopic thyroid cancer surgery, hypoparathyroidism, prophylactic central neck dissection

## Abstract

**Objective:**

To evaluate the value of refined extracapsular anatomy combined with carbon nanoparticle suspension tracing technology for protecting parathyroid function and the thoroughness of lymph node dissection in the central region during endoscopic thyroid cancer surgery.

**Patients and methods:**

Retrospective clinical data analysis was performed on 108 patients who underwent endoscopic thyroid cancer surgery at the First Affiliated Hospital of Wannan Medical College (Yijishan Hospital) from November 2019 to November 2022. Before surgery, thyroid function tests, color Doppler ultrasounds and neck-enhanced CT scans were performed on all patients. Cytopathological diagnosis obtained *via* ultrasound-guided fine-needle aspiration served as confirmation for the primary diagnosis. It was determined whether to perform a total thyroidectomy or a hemithyroidectomy (HT) together with preventive unilateral (ipsilateral) central neck dissection. Follow-up times were 1 to 34 months.

**Results:**

Transient neuromuscular symptoms were present in 3.70% (4/108) cases, with no permanent neuromuscular symptoms or permanent hypoparathyroidism. Regarding transient hypoparathyroidism, the patients recovered after three months and did not need long-term calcium supplementation. The number of harvested LNs (mean± SD) was 5.54 ± 3.84, with ≤5 in 57.41% (62/108) and >5 in 42.59% (46/108) cases. The number of patients with metastatic LNs was 37.96% (41/108), with ≤2 in 65.85% (27/41) and >2 in 34.15% (14/41) cases.

**Conclusions:**

Fine extracapsular anatomy combined with carbon nanoparticle suspension tracing is effective in endoscopic thyroid cancer surgery. It can improve the thoroughness of prophylactic central neck dissection and recognition of the parathyroid gland and avoid parathyroid injury and other complications to effectively protect parathyroid function.

## Introduction

According to the global IARC tumor registration report on five continents, the incidence of thyroid cancer increased sharply from 1973 to 2002, and the incidence rate in women was three times higher than in men (1). According to statistics from the China Cancer Center, the age-standardized incidence rate of thyroid cancer in China was 168.08/100000 in 2012, with the age-standardized incidence rate in women being 169.4/100000 (2). The incidence rate of thyroid cancer in China is increasing at an annual rate of 5.92%, which is 4% higher than the global incidence rate. Moreover, the incidence in big cities is higher than that in rural areas. Overall, the incidence rate in females is higher than that in males (3–5). Due to the development of urbanization and improvement of economic conditions, women have higher esthetic requirements. To avoid neck scars, endoscopic thyroid surgery is increasingly carried out to improve the cosmetic effect (6, 7). Protecting the parathyroid gland versus the thoroughness of lymph node dissection in the central region during endoscopic thyroid cancer surgery has remained controversial. When the parathyroid gland is injured, parathyroid hormone levels may decrease, which may cause the patient to experience various symptoms, including perioral numbness and convulsions. Incomplete dissection of lymph nodes in the central region is an important reason for local recurrence after the operation. Indeed, a recent retrospective analysis of 399 patients showed that thorough lymph node dissection in the central region can significantly increase the disease-free survival (DFS) after thyroid cancer surgery, especially with respect to occult lymph node metastasis, which is an important factor affecting prognosis (8). Moreover, intraoperative use of indocyanine green and carbon nanoparticle suspensions as tracers can protect the parathyroid gland and increase the thoroughness of lymph node dissection in the central region (9). Our department uses a carbon nanoparticle suspension to trace lymph nodes during surgery in an effort to improve the thoroughness of lymph node dissection in the central area, protect parathyroid function, and reduce postoperative complications and local recurrence. As a new type of lymphatic tracer, carbon nanoparticles (CNs) have an active movement mechanism. CNs are phagocytosed by macrophages and then enter the lymphatic capillaries and accumulate in lymph nodes, such that the thyroid gland and lymph nodes in the drainage area quickly become blackened according to the order of thyroid lymphatic drainage (10, 11). As there are no communicating lymphatic vessels between the thyroid tissue and the parathyroid gland, the thyroid tissue and surrounding lymph nodes become stained black after CNs are injected into the thyroid tissue, whereas the parathyroid gland remains unstained. This is called the “negative parathyroid imaging effect” and is used to protect the parathyroid gland (12). In this study, we sought to assess the application value of refined extracapsular anatomy combined with a CNs suspension for protecting parathyroid function and lymph node dissection in the central region during endoscopic surgery for thyroid cancer.

## Materials and methods

### Patient enrollment

Clinical data for 108 patients with thyroid cancer who underwent surgery at the First Affiliated Hospital of Wannan Medical College from November 2019 to November 2022 were analyzed retrospectively. All patients underwent thyroid function measurement, color Doppler ultrasound examination and neck enhanced CT examination before the surgery. Diagnoses were confirmed by cytopathology based on fine-needle aspiration under ultrasound guidance.

### Criteria for inclusion

(1) No history of neck surgery; (2) normal parathyroid gland function; (3) papillary thyroid carcinoma; (4) no invasion of the thyroid envelope; (5) enhanced CT showing no lateral cervical lymph node metastasis; and (6) no abnormalities in preoperative routine blood tests, blood coagulation function tests, chest CT, fiberoptic laryngoscopy, liver and kidney function tests or electrolyte examination.

### Criteria for exclusion

(1) History of neck radiotherapy; (2) hyperthyroidism; (3) Hyperparathyroidism; (4) medullary thyroid carcinoma or anaplastic thyroid cancer; (5) thyroid tumor recurrence.

### Surgical procedure and CN suspension injection

**Figure 1:**
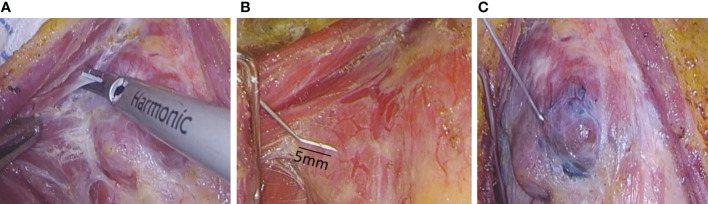
Refined extracapsular anatomy and injection of carbon nanoparticles suspension. **(A)** refined extracapsular anatomy **(B)** The needle tip of the syringe is bent by 5mm **(C)** slow injection of carbon nanoparticles suspension.

**Figure 2:**
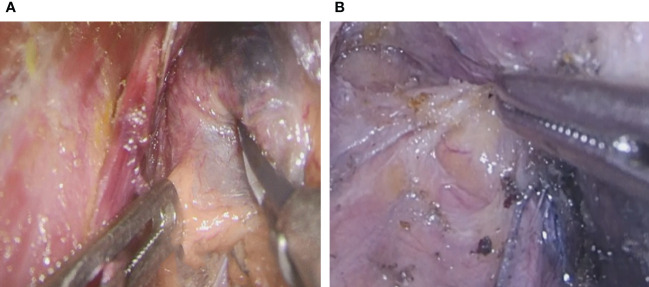
Parathyroid gland expose. **(A)** Inferior parathyroid gland **(B)** Superior parathyroid gland.

**Figure 3:**
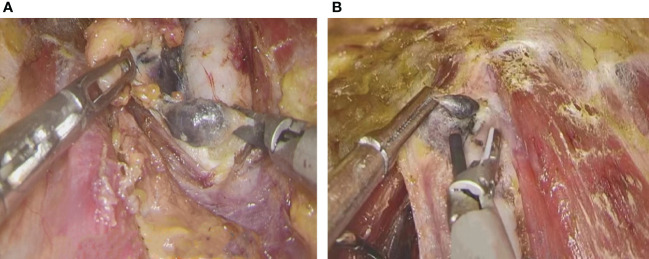
Resection of lymph nodes. **(A)** resection of lymph nodes on pre-tracheal **(B)** resection of anterior laryngeal lymph nodes.

All operations were performed by a professional and experienced thyroid surgeon. Endoscopic radical thyroidectomy was performed *via* the oral vestibule, the axillo-breast approach, and the areolar approach. The thyroid gland was exposed, and the integrity of the thyroid surgical capsule was maintained according to the principle of membrane anatomy (**Figure 1A**). CNs (0.1-0.2 ml) were drawn into a 1-mL skin test syringe, and the thyroid gland was punctured at a depth of 5 mm (**Figure 1B**). The CNs were slowly injected into the tissue around the gland tumor on the affected side under the true capsule (**Figure 1C**). During the injection, it is necessary to avoid the tumor and blood vessels and to slowly inject after drawing back blood to prevent the CN suspension from entering the blood vessels; the injection volume was 0.1-0.2 mL. After the injection, a gauze strip was pressed for a moment to prevent CN overflow, as overflowing CNs easily blacken the surgical field, affecting the operation. The CNs diffuse into the thyroid tissue within 3 min. We also found some lymph nodes stained black at the central compartment of the neck, though the parathyroid glands were not stained. Endoscopic thyroidectomy and prophylactic lymph node dissection were performed in the central region (13). Due to the negative development of CNs, we carefully looked for the inferior parathyroid gland and kept it in place to avoid damaging the parathyroid vessels (**Figure 2A**). The anterior branch of the superior thyroid artery was slowly coagulated with an ultrasonic knife, and we operated close to the gland to avoid damaging the external branch of the superior laryngeal nerve. The posterior branch of the superior thyroid artery was maintained as much as possible; that is, the “off hat method” was used for the superior thyroid. The superior parathyroid gland can generally be clearly identified and retained *in situ* (**Figure 2B**) (14, 15). Lymph node dissection in the central area was performed after thyroid cancer was confirmed by the frozen section procedure during the operation. As CNs track the lymph nodes, they become stained black, which is conducive to dissection of lymph nodes in the central area (**Figures 3A, B**).

### Observations

(1) The time needed for the operation, total amount of postoperative drainage, drainage time and postoperative hospital stay were noted.(2) The number of cases with transient hoarseness and permanent hoarseness was assessed. (3) The number of cases with transient hypocalcemia neuromuscular symptoms and permanent neuromuscular symptoms and the incidence of transient and permanent hypoparathyroidism were also evaluated. (4) The number of lymph nodes removed and the number of positive lymph nodes in the central area were recorded.

### Statistical analysis

SPSS 22.0 software (Chicago, IL, USA) was used for statistical analyses. Continuous variables are expressed as mean ± standard deviations (SDs). A P value less than 0.05 was considered statistically significant.

## Results

### Characteristics of patients

A total of 108 patients underwent successful endoscopic thyroidectomy. The demographics are shown in **Table 1**. There were 4 males and 104 females, ages ranged from 15 to 54 years, with an average age of 30.98 ± 6.38 years. Height (cm) (mean ± SD) of the patients was 162.16 ± 4.43, weight (kg) (mean ± SD) was 58.54 ± 8.70, and body mass index (BMI) (mean ± SD) was 22.39 ± 3.50. Tumor sites were as follows: middle/upper portion 31, middle 31, middle/lower portion 46.

**Table 1:** Clinical data of patients.

Characteristics of patients (n = 108)	Absolute NO.	Relative %
Age, years (mean ± SD)	30.98±6.38	
Age(years)		
≤55	108	100%
>55	0	0%
Gender		
Male	4	3.70%
Female	104	96.30%
Height (cm) (mean ± SD)	162.16±4.43	
Height (cm)		
≤160	47/108	43.52%
>160	61/108	56.48%
Weight (kg) (mean ± SD)	58.54±8.70	
Weight (kg)		
≤60	75	69.44%
>60	33	30.56%
BMI (mean ± SD)	22.39±3.50	
≤25	92	85.19%
>25	16	14.81%
Tumor site		
Middle /Upper portion	31/108	28.70%
Middle	31/108	28.70%
Middle / lower portion	46/108	42.60%
Tumor Side		
left	48/108	44.44%
right	60/108	55.56%

SD, standard deviation; BMI, body mass index.

### Carbon nanoparticles improves the protective effect on the parathyroid gland

Data for the patient operations are shown in **Table 2**. The operation paths were as follows: 32 cases of the oral vestibule, 56 cases of the axillo-breast approach and 20 cases of the areolar approach. Six patients underwent total thyroidectomy + central neck dissection (CND) and 102 hemithyroidectomy +CND. Postoperative transient hoarseness occurred in 3/108 (2.77%) patients, with no cases of permanent hoarseness. Only 3 patients experienced hoarseness and recovered after one month of medical therapy. Transient neuromuscular symptoms occurred in 4/108 (3.70%) patients; there were no permanent neuromuscular symptoms. No permanent hypoparathyroidism occurred. Those who experienced transient hypoparathyroidism recovered after three months, with no need for long-term calcium supplementation. CNs can significantly prevent parathyroid injuries. There was no recurrence in our cohort. The operation time was 156.94 ± 44.48 minutes, and the total postoperative drainage was 149.69 ± 53.05 ml. The postoperative drainage time was 3.46 ± 0.65 day. The postoperative hospital stay time was 3.49 ± 0.65 day.

**Table 2:** Operation and post operation condition.

Characteristics of patients (n = 108)	Absolute NO.	Relative %
Operation path		
Transoral vestibule	32	29.62%
Transthoracic breast	56	51.85%
Transaxillary	20	18.53%
Extent of surgery		
total thyroidectomy +CND	6/108	5.56%
hemi thyroidectomy +CND	102/108	94.45%
Postoperative hoarseness		
Transient	3/108	2.77%
Permanent	0	0%
Neuromuscular symptoms		
Transient	4/108	3.70%
Permanent	0	0%
Hypoparathyroidism		
Transient	5/108	4.62%
Permanent	0	0%
Recurrence	0	0%
Operation time (mins, mean± SD)	156.94±44.48	
Total postoperative drainage (ml, mean±SD)	149.69±53.05	
Postoperative drainage time (day, mean±SD)	3.46±0.65	
Postoperative hospital stay time (day, mean±SD)	3.49±0.65	

CND, central neck dissection; SD, standard deviation.

### Carbon nanoparticles facilitate harvesting of lymph nodes

The postoperative pathological characteristics were shown in **Table 3**. Mean tumor size was 0.72 ± 0.28 cm, 88.89%(96/108) patients with a tumor ≤1 cm and 11.11%(12/108) with a tumor >1 cm. The average numbers of harvested LNs were 5.54 ± 3.84. There were 57.41% (62/108) patients have ≤5 LNs and 42.59% (46/108) have >5 LNs. There were 37.96% (41/108) patients have metastatic LNs. The average numbers of metastatic LNs were 0.91 ± 1.74. There were 65.85% (27/41) patients have ≤2 metastatic LNs and 34.15% (14/41) have >2 metastatic LNs. For all the 108 patients, Histological grading was grade I, and pathological TNM staging was stage I.

**Table 3:** Characteristics postoperative pathological.

Characteristics of patients (n = 108)	Absolute NO.	Relative %
Tumor size(cm)(mean ± SD)	0.72±0.28	
≤1	96/108	88.89%
>1	12/108	11.11%
Pathological type		
Papillary carcinoma	108/108	100%
Other types	0	0%
Numbers of harvested LNs (Pieces mean± SD)	5.54±3.84	
≤5	62/108	57.41%
>5	46/108	42.59%
metastatic LNs (Pieces mean± SD)	0.91±1.74	
≤2	27/41	65.85%
>2	14/41	34.15%
Numbers of patients with metastatic LN		
LN positive	41/108	37.97%
LN negative	67/108	62.03%
Histological grading		
I	108/108	100%
II-III	0	0%
Pathological staging		
I	108/108	100%
II	0	0%
III	0	0%

LNs, lymph nodes; SD, standard deviation.

## Discussion

Currently, the majority of young patients have accepted endoscopic thyroid surgery because of its advantages, such as minimal invasiveness and scarless. This approach has the advantages of enlarging the surgical area and high resolution of the surgical field of view. There were only 4 males but 104 females in our study, and average age was 30.98 ± 6.38 years old. May be young women have high requirements for beauty, and there is an advantage of scarlessness in endoscopic radical thyroidectomy.

Whether it can effectively protect the parathyroid gland and completely remove the lymph nodes in the central region has been the focus of controversy. In general, endoscopic lymph node dissection is difficult without navigation for tracking the lymph nodes. Therefore, searching for effective materials for lymph node navigation and tracking is a hot research topic. As a new lymphatic tracer, CNs have the advantages of rapid staining, long duration and clear lymphatic tracing, and they have been applied in both open thyroid cancer surgery and endoscopic thyroid cancer surgery. CNs have an active movement mechanism, whereby they are engulfed by macrophages and then enter the lymphatic capillaries, accumulating in the lymph nodes. Thus, the thyroid gland and lymph nodes in the drainage area quickly become blackened according to the order of thyroid lymphatic drainage (10, 11). CNs can help surgeons to distinguish between lymph nodes and fat particles. Some studies have shown that the small lymph nodes (diameter<5 mm) in the central area and the relatively hidden lymph nodes are difficult to identify during the cleaning process and are easily ignored. With CN staining and the magnifying effect of endoscopy, the operator can easily find and remove the small lymph nodes in this area, significantly promoting the thoroughness of lymph node cleaning (16, 17). The number of harvested LNs was 5.54 ± 3.84 in our study. Compared with a retrospective cohort study, 114 PTC patients undergoing bilateral axillo-breast approach robotic thyroidectomy (BABART) were enrolled and divided into CNs group (n=64) and control group (n=50). The mean number of retrieved central lymph node was significantly higher in the CNs group than in the control group (9.48 ± 4.88 vs. 5.40 ± 2.67, P<0.001) (18). The other prospective randomized study, three hundred two consecutive early-stage thyroid cancer patients eligible for endoscopic thyroidectomy *via* bilateral areola approach (ETBAA) were recruited and divided into two group: a carbon nanoparticles group (n=152) and a control group (n=150). The total number of dissected lymph nodes was 1059 in the carbon nanoparticles group and 872 in the control group (P=0.00) (19). The applying of carbon nanoparticle can help to detect lymph nodes and increase the number of lymph nodes visualized and preserved.

**Figure 4:**
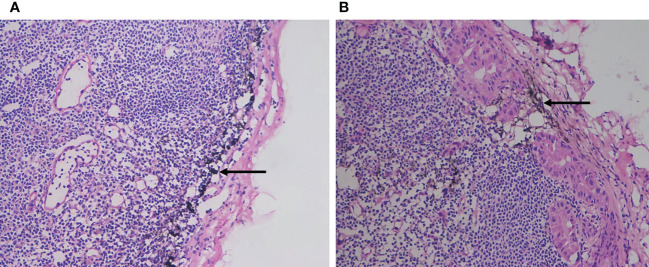
The tracing effect of CNs. **(A)** The carbon nanoparticles were located in the marginal sinus of the lymph node without metastasis of the tumor **(B)** The carbon nanoparticles were located in the marginal sinus of the lymph node where the tumor had metastasis.

**Figure 5:**
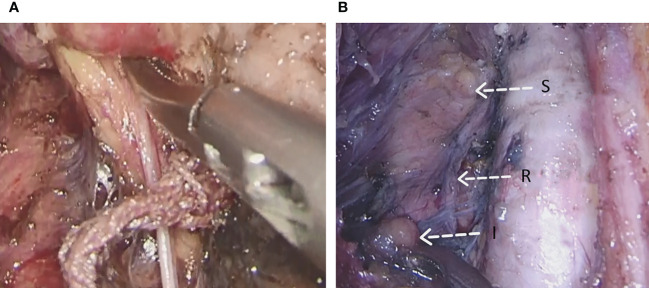
Laryngeal nerve and parathyroid gland protecting. **(A)** establishment of recurrent laryngeal nerve tunnel to protecting **(B)** Surgical field after thyroidectomy (S, Superior parathyroid gland; R, Recurrent laryngeal nerve; I, Inferior parathyroid gland).

In addition, the tracing effect of CNs has a guiding role for pathologists to take lymph nodes after surgery, reducing the impact of human factors on the number of lymph nodes (**Figures 4A, B**) (20). In preventive lymph node dissection in the central region, CNs can be used as a tracer to make the parathyroid gland easy to identify, avoiding the risk of injury to the recurrent laryngeal nerve and parathyroid gland (**Figures 5A, B**) (21). In fact, parathyroid gland injury can lead to a decrease in parathyroid hormone, and patients will have symptoms such as perioral numbness and hypocalcemia convulsions. If permanent hypoparathyroidism occurs, long-term oral or intravenous calcium supplementation is needed, which affects quality of life (22, 23). When CNs are injected into the thyroid tissue, the surrounding lymph nodes stain black, though the parathyroid gland does not. When the thyroid gland is removed, fine envelope dissection is used to protect the nonblack-stained tissue as much as possible, reducing the risk of parathyroid damage during lymph node dissection of thyroid cancer (24). As there are no lymphatic vessels communicating between the thyroid tissue and the parathyroid gland, the thyroid tissue and surrounding lymph nodes are stained black after CNs are injected into the thyroid tissue, whereas the parathyroid gland remains unstained. This “negative parathyroid imaging effect” achieves localization, recognition and protection of the parathyroid gland and reduces the incidence of postoperative hypothyroidism (12). However, it has been reported that through the whole areola approach in endoscopic thyroid cancer surgery, the use of CNs cannot significantly enhance protection of the parathyroid glands, especially the lower parathyroid gland, and cannot significantly benefit protection of the parathyroid glands or lymph node dissection (25, 26). In our study, 2.77% (3/108) patients suffered transient postoperative hoarseness and 4.62% (5/108) patients appeared transient hypoparathyroidism. According to a meta-analysis, the median incidence of transient and permanent hypoparathyroidism was 27% (19%-38%) and 1% (0-3%) respectively (27). Another study based on the SEER-Medicare linked database shows that 11% of the cases need calcium and vitamin D supplementation for hypoparathyroidism (28). Furthermore, the immune response caused by residual CNs in the thyroid bed may increase tissue inflammation and edema, thus reducing the venous flow of the parathyroid gland, which has a negative impact on parathyroid gland function (29).

There are also some limitations of our study. Firstly, all the 108 patients received endoscopic thyroid cancer surgery and the CNs was used during operation. And we don’t get the data of patients without using CNs. However, there are many similar research in the literature, and our data ware basically consistent with others. Secondly, many postoperative symptoms were collected, but little objective indicator, like serum PTH and Ca^2+^ level, was tested. Mainly because that we didn’t test PTH or Ca^2+^ level after surgery routinely in our center, unless bilateral thyroidectomy was performed or patients with overt symptom. And we are preparing to conduct a prospective study in the near future.

## Conclusion

In summary, the application of refined extracapsular anatomy combined with a CN suspension tracing technology has a definite effect in endoscopic thyroid cancer surgery, improving the thoroughness of preventing lymph node dissection in the central region, enhancing recognition of the parathyroid gland, and avoiding parathyroid injury or accidental resection as well as other complications, thus effectively preserving parathyroid function.

## Data availability statement

The raw data supporting the conclusions of this article will be made available by the authors, without undue reservation.

## Ethics statement

The studies involving human participants were reviewed and approved by the Ethics Committee of the (Yijishan Hospital) First Affiliated Hospital of Wannan Medical College. The patients/participants provided their written informed consent to participate in this study. Written informed consent was obtained from the individual(s) for the publication of any potentially identifiable images or data included in this article. The study protocol was approved by the Ethics Committee of the (Yijishan Hospital) First Affiliated Hospital of Wannan Medical College. Informed consent was obtained from all individual participants included in the study.

## Author contributions

The article was mainly written by ZW and RS. ZW, HB, YX, and ZB, these authors contributed equally to the study, ZW, HB, YX, EL, XS and ZB helped with data analysis and paper editing. YW, ZW, ZY, and CY did an operation study. The whole study was instructed by BC, YW and RS. All authors contributed to the article and approved the submitted version.
